# Validation of a Portable Ionized Calcium Detection Device and Changes in the Ionized-to-Total-Calcium Ratio in the Blood of Postpartum Holstein Cows

**DOI:** 10.3390/ani15020136

**Published:** 2025-01-08

**Authors:** Zhongming Cao, Yan Zhao, Bo Zhang, John P. Kastelic, Mengjie Hu, Jia Cheng, Mingchao Liu, Jian Gao

**Affiliations:** 1 College of Veterinary Medicine, China Agricultural University, Beijing 100193, China; caozhongming296@outlook.com (Z.C.); humengjie2000@163.com (M.H.); 2 College of Veterinary Medicine, Hebei Agricultural University, Baoding 071001, China; zhaoyan200012@163.com (Y.Z.); chengjia@hebau.edu.cn (J.C.); liumingchao@163.com (M.L.); 3 Modern Dairy (Group) Co., Ltd., Maanshan 243000, China; zhangbo_2009-6-16@163.com; 4 Faculty of Veterinary Medicine, University of Calgary, Calgary, AB T2N 4Z6, Canada; jpkastel@ucalgary.ca

**Keywords:** hypocalcemia, ionized calcium, total calcium, cattle, milk cows

## Abstract

The calcium requirements of dairy cows increase dramatically soon after calving, and many cows have subclinical hypocalcemia, adversely affecting health and performance. Traditional laboratory tests for calcium are complex and not easily adapted to rapid point-of-care applications. The objectives were to evaluate a portable iCa testing device, Horiba LAQUAtwin Ca-11C, for measuring ionized calcium (iCa) in the whole blood of dairy cows and to investigate the iCa-to-total-calcium (tCa) ratio in blood collected from dairy cows within 9 days after calving. This study was conducted on two large dairies in northern China. First, blood samples were collected from 246 cows within 3 days after calving, and whole-blood iCa concentrations were measured with a Horiba LAQUAtwin Ca-11C and Abbott i-STAT 1. In addition, 885 blood samples were collected from 102 dairy cows between calving and 9 days postpartum, with iCa concentrations measured using a Horiba LAQUAtwin Ca-11C and tCa concentrations determined by a fully automated biochemical analyzer. The Horiba LAQUAtwin Ca-11C and Abbott i-STAT 1 had significant consistency in the measurement of ionized calcium concentration in whole blood (the frequency of differences between the measured values was within ±20% of the average, reaching 95.53%), and the measurement results of the two instruments were correlated (Deming regression analysis R^2^ = 0.87). This implied the potential application of the Horiba LAQUAtwin Ca-11C as a simplified device for measuring iCa in dairy farms for on-site testing. Within the first 9 days after calving, the ratio of ionized calcium to total calcium fluctuated but overall remained within the range of 44.2 to 47.22%. Furthermore, significant variations in the ratio among individual cows indicated that individual differences and physiological states of cows affected calcium metabolism. This study identified the potential to use the Horiba LAQUAtwin Ca-11C for point-of-care testing on dairy farms. Nevertheless, the impacts of factors such as health status and individual cow differences on the ratio of ionized calcium to total calcium still require further investigation.

## Simple Summary

The objectives of this study were to (1) compare a portable calcium testing device, Horiba LAQUAtwin Ca-11C, and an Abbott i-STAT 1, for measuring ionized calcium (iCa) concentrations in the whole blood of dairy cows and (2) investigate trends in the iCa-to-total-calcium (tCa) ratio after calving. Blood samples were collected from 246 cows within 3 days after calving, and the two instruments had close agreement in their measurements (Deming regression analysis R^2^ = 0.87). Blood samples (n = 885) were collected from 102 cows between calving and 9 days postpartum; the iCa-to-tCa ratio fluctuated but remained within a modest range. However, there were significant differences among individual cows, suggesting health status and individual variations influenced calcium metabolism. This study verified the potential use of Horiba LAQUAtwin Ca-11C for point-of-care analysis on dairy farms and highlighted the need for further investigations into factors affecting calcium metabolism.

## 1. Introduction

Calcium has key roles in various physiological processes in animals, including immune function, blood coagulation, nerve impulse conduction, and muscle contraction [1]. Cows synthesize colostrum during late pregnancy, with increasing production of colostrum and subsequently milk in the first few days after calving, creating large demands for calcium. Consequently, >50% of postpartum dairy cows have a significant decrease in blood calcium concentrations, leading to hypocalcemia [2,3]. During this period, calcium mobilization is critical to maintaining plasma Ca^2+^ reserves to meet requirements for colostrum and milk [4]. However, ~5 to 10% of high-yielding cows develop clinical signs of hypocalcemia (milk fever) after calving [5]. Furthermore, 30 to 60% have subclinical hypocalcemia [5,6] (total serum calcium ≤ 2.1 mmol/L), increasing risks of mastitis and other secondary conditions [7].

Calcium in the blood of dairy cows exists in three forms: ~50% as free ions, 40% bound to proteins, and 10% complexed with anions such as lactate, citrate, inorganic phosphate, or bicarbonate. These three components balance each other [8], but only the ionized form is directly available for maintaining calcium homeostasis [9].

Measuring blood calcium concentrations in cows without clinical signs is a key tool for detecting subclinical hypocalcemia [2,10]. However, since only ionized calcium (iCa) is biologically active, iCa concentrations are more meaningful than total calcium (tCa) concentrations [11]. In human medicine, plasma iCa concentrations have been widely used to monitor physiological status [8]. In contrast, in veterinary medicine, tCa is usually measured, as it is less technically challenging. Although tCa can be measured several hours after sampling, or in frozen–thawed serum samples, iCa must be measured immediately from a gas-tight container because the proportion of protein-bound calcium decreases with increasing pH, as changes in blood gases can affect iCa concentrations [5,12,13]. For technical reasons, traditional laboratory biochemistry analyzers are not well suited for rapid point-of-care testing on dairy farms. Therefore, developing portable, rapid, and accurate tools for measuring iCa concentrations is crucial.

The Horiba LAQUAtwin Ca-11C, a commercially available device for point-of-care measurement of whole-blood iCa in dairy cows, was provisionally validated [14]. Our objective was to compare this device and an Abbott iSTAT-1, the gold standard for cow-side testing of blood iCa concentrations. The i-STAT 1 is a reliable detection tool for measuring ionized calcium (iCa) concentrations in cow serum. However, its application in dairy farms is restricted by strict operating temperature requirements (range of 17–30 °C), high cost, and long detection time. In contrast, the Horiba LAQUAtwin Ca-11C offers more convenient operating conditions, a shorter operation time, and reduced sensitivity to external temperature; however, its application for large-scale detection of hypocalcemia in dairy cows has not been well studied. In addition, we aimed to determine the whole-blood iCa-to-tCa ratio in postpartum cows. This study provided new data for evaluating potential applications of the Horiba LAQUAtwin Ca-11C device in disease prevention and health management on dairy farms.

## 2. Materials and Methods

Blood collection and animal handling procedures were reviewed and approved by the Experimental Animal Welfare and Animal Experiment Ethical Review Committee of China Agricultural University (protocol code AW71214202-2-01).

### 2.1. Dairy Farms and Cows

This experiment was conducted on two large dairies (Farms A and B, respectively) in Hohhot, Inner Mongolia, China. Farm A has a lactating cow inventory of ~2500 cows, whereas Farm B has ~5000 cows. On both farms, all cows are Holstein, fed a total mixed ration, milked three times a day, and produce an annual average of ~12 tons of milk per cow. In addition, both farms feed an anionic diet during the periparturient period, and the incidence of clinical hypocalcemia is <2%. Blood samples were collected from live animals, and an official exemption letter was received from the Experimental Animal Welfare and Animal Experiment Ethical Review Committee of China Agricultural University.

Both farms add anionic salts to the diets of cows during the prepartum period to prevent milk fever. Additionally, they oat hay use during the prepartum period to reduce concentrations of potassium and sodium ions in the diets. The experimental cows (246 and 102, respectively) within the two dairy farms were all individually reared in semi-open postpartum care barns. Dairy cows received daily routine postpartum care and pen inspections to ensure access to feed and drinking water, to minimize stress, and to facilitate blood collection and blood calcium testing.

In Farm A, proportions of the third-, fourth-, and fifth (or more)-parity were 43.9, 41.5, and 14.6%, respectively. In Farm B, proportions of first-, second-, and third (or more)-parity were 24.8, 48.6, and 2.66%.

### 2.2. Experimental Design

#### 2.2.1. Trial 1: Comparing a Horiba LAQUAtwin Ca-11C and an Abbott iSTAT-1 for Measuring iCa Concentrations in Whole Blood

**Figure 1:**
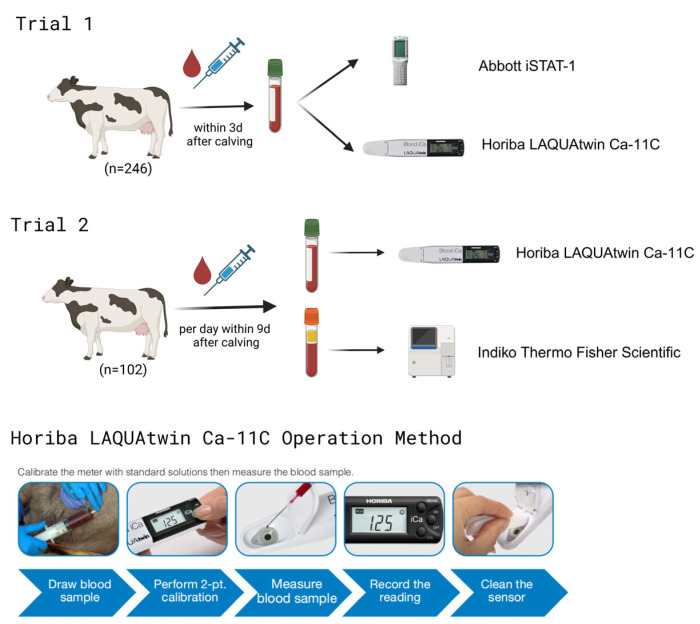
Flowchart of experimental procedures for Trials 1 and 2, and operation diagram of Horiba LAQUAtwin Ca-11C. The operation part of the content was derived from the Horiba LAQUAtwin Ca-11C Operating Manual.

To compare iCa concentrations between these two devices, within 3 days after calving on Farm A, a blood sample was collected from the coccygeal vessels of 246 cows into a tube containing heparin. Within 30 min after blood collection, iCa concentrations were measured synchronously at cow-side using both a Horiba LAQUAtwin Ca-11C (LAQUAtwin Ca-11C, HORIBA Advanced Techno, Kyoto, Japan) and an Abbott iSTAT-1 (Abbott Point of Care, Abbott Laboratories, Chicago, IL, USA). None of the cows had any clinical signs or indications of any abnormality. The specific process can be referred to in Figure 1.

#### 2.2.2. Trial 2: Association Between Blood iCa and tCa in Postpartum Dairy Cows

To assess associations between iCa and tCa in the blood of postpartum dairy cows, 885 blood samples were collected from 102 cows on Farm B. Relative to calving, blood samples were collected from coccygeal blood vessels of 102 cows at 0 h (n= 94), day 1 (n = 89), day 2 (n = 84), day 3 (n = 86), day 4 (n = 84), day 5 (n = 90), day 6 (n = 84), day 7 (n = 91), day 8 (n = 89), and day 9 (n = 94). In total, 885 blood samples were collected. Whole-blood iCa (iCa) concentrations were measured with Horiba LAQUAtwin Ca-11C, whereas serum tCa (tCa) concentrations were measured using Indiko Thermo Fisher Scientific (Thermo Scientific™ Indiko™, Thermo Fisher Scientific, Waltham, MA, USA) to determine the association between machines and the iCa/tCa ratio. The specific process can be referred to in Figure 1.

### 2.3. Collection of Blood Samples

In Farm A, blood samples from all 284 cows were collected at ~0800 every day, whereas on Farm B, 0 h blood samples from 102 cows were collected immediately after calving, and blood samples from 1 to 9 days postpartum were collected at 0800 or 2000 daily. Throughout the trial, the personnel performing blood sample collection and testing was kept constant, and the ambient temperature was 18 to 32 °C with <80% relative humidity.

Blood samples were collected from coccygeal vessels via venipuncture using a 24 mm, 20 gauge, beveled-mouth blood collection needle. To ensure that samples were collected under anaerobic conditions, the needle was first withdrawn from the blood collection tube and then from the blood vessel to prevent air from entering the blood sample. For determining iCa, blood was collected into a 10 mL lithium heparin vacuum blood collection tube (10 IU/mL, KWS blood collection tube). Another blood sample was collected into a 10 mL plain vacuum blood collection tube, and after allowing 30 min for clot formation, it was centrifuged at 1500× *g* for 10 min, and the serum was recovered and stored at −20 °C and used for a tCa assay within 1 week.

### 2.4. Calcium Measurements

Horiba LAQUAtwin Ca-11C operation: After powering on the device, a secondary calibration was performed using 1.25 and 2.5 mmol/L standard solutions. Then, 0.3 mL of a blood sample was added to the sensor, the “Measure” button was pressed, and the results were recorded once displayed. Abbott i-STAT 1 Operation: A blood sample was slowly added to the CG8+ cartridge using a rubber-tipped burette. Subsequently, the cardholder was inserted into the device slot, and the results were recorded once displayed. Indiko Thermo Fisher Scientific operation: Serum was added into the fully automated biochemical analyzer, and the test was performed directly. Device information is summarized in Table 1.

This study primarily compared the suitability of Abbott iSTAT-1 (Abbott Point of Care, Abbott Laboratories, Chicago, IL, USA), Indiko Thermo Fisher Scientific (Thermo Scientific™ Indiko™, Thermo Fisher Scientific, Waltham, MA, USA), and Horiba LAQUAtwin Ca-11C (LAQUAtwin Ca-11C, HORIBA Advanced Techno, Kyoto, Japan) for the detection of ionized calcium in dairy cow blood. Specific discrepancies among the three machines are presented in Table 1.

### 2.5. Statistical Analyses

Descriptive statistics were performed using software (IBM SPSS Statistics for Windows, Version 21.0, IBM Corp., 2012, Armonk, NY, USA). Cumulative sum tests of linearity (from Passing and Bablok regressions, Experiments 1 and 2), Bland–Altman plots, and Deming regressions (Experiment 1) were performed. The analysis conducted by the i-STAT was considered the reference method, and the use of the Horiba LAQUAtwin Ca-11C served as the test method. The iCa^2+^ concentrations measured by the Horiba LAQUAtwin Ca-11C were regressed on tCa^2+^ concentrations measured by the Indiko Thermo Fisher Scientific (n = 885) using GraphPad Prism version 7.03 (GraphPad Software, La Jolla, CA, USA) to determine linear associations, generating a mean, standard deviation, and coefficient of variation (Experiment 2). Deming regression analyses were performed. Pearson’s correlation and linear regression analyses were used in statistical analyses, and the test of significance was set at *p* < 0.05. Data were graphed using GraphPad Prism version 7.03 (GraphPad Software, La Jolla, CA, USA).

## 3. Results

### 3.1. Comparison of iCa Values from Horiba LAQUAtwin Ca-11C and Abbott i-STAT 1

**Figure 2:**
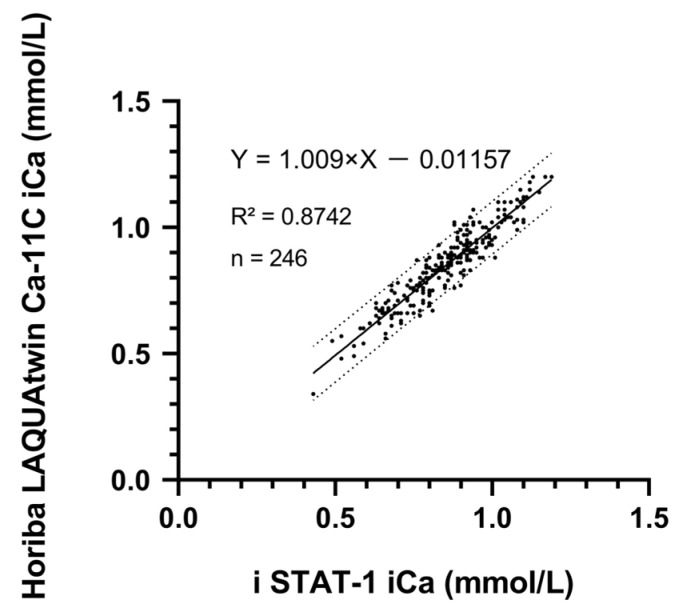
Deming regression analysis plots of Horiba LAQUAtwin Ca-11C versus i-STAT 1 whole-blood iCa (iCa) concentration under bovine parabiotic assay conditions. The dashed and solid lines represent the regression line Y = 1.009X − 0.01157, respectively, where the intercept is −0.01157 (95% confidence interval: −0.05336 to 0.03022) and the slope is 1.009 (95% confidence interval: 0.970 to 1.080).

The iCa concentrations were determined in 246 blood samples. Horiba LAQUAtwin Ca-11C measurements were plotted on the y-axis whereas data from i-STAT 1, as the reference method, were plotted on the x-axis. Deming regression analyses had an intercept of −0.100 (95% confidence interval of −0.05336 to 0.03022) and a slope of 1.009 (95% confidence interval of 0.970 to 1.080). Thus, there was no proportionality bias between i-STAT 1 and the iCa values detected by Horiba LAQUAtwin Ca-11C under ambient conditions for the cow-side test, as the 95% confidence intervals for the intercepts and slopes contained 0 and 1, respectively. Furthermore, the concordance correlation coefficient was 0.8742, indicating a significant concordance between the two (Figure 2). In addition, regarding residual analysis of the Horiba LAQUAtwin Ca-11C with i-STAT 1, the mean bias of the Horiba LAQUAtwin Ca-11C readings was within −0.04.

**Figure 3:**
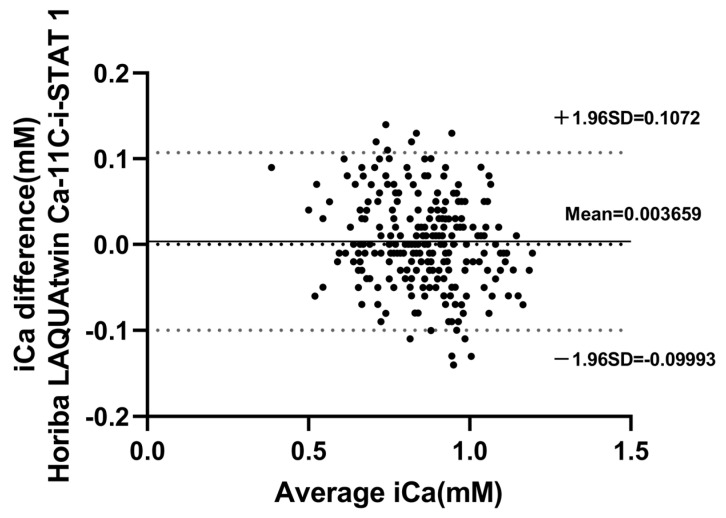
Bland–Altman plots of Horiba LAQUAtwin Ca-11C versus i-STAT 1 whole-blood iCa (iCa) concentration under bovine parabiotic assay conditions. The solid line indicates the mean difference between the two methods, and the dashed line indicates the 95% confidence interval.

The Bland–Altman plot further validated the good agreement, as iCa values detected by the two instruments were considered “compatible” since measured values differed at a frequency of 95.53% in the range of ±20% of the mean value (235/246, significantly > 75%) (Figure 3).

### 3.2. Relationship of Whole-Blood iCa Values with Indiko Thermo Fisher Scientific Serum tCa Values Using the Horiba LAQUAtwin Ca-11C Assay

**Figure 4:**
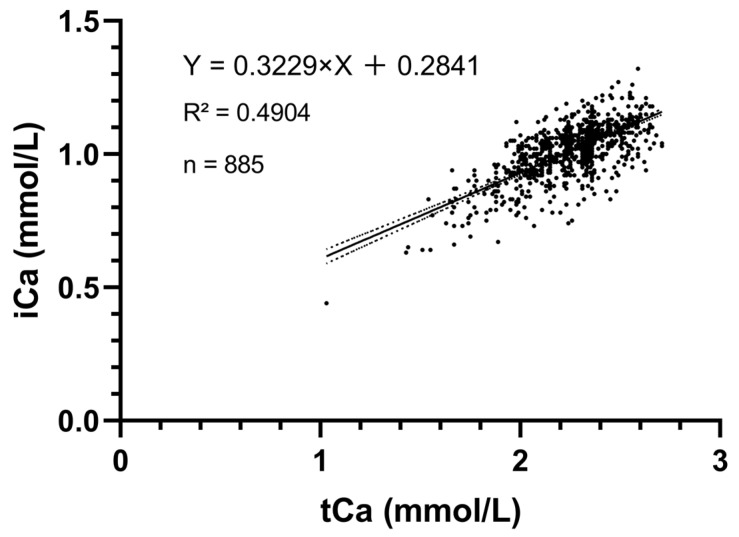
Deming regression plot of whole-blood iCa (iCa) using Horiba LAQUAtwin Ca-11C bovine paracentesis versus total serum calcium (tCa) concentrations using Indiko Thermo Fisher Scientific. The regression equation was Y = 0.3229X + 0.2841 with an intercept of 0.2841 (95% confidence interval: 0.2351 to 0.3332) and a slope of 0.3229 (95% confidence interval: 0.3011 to 0.3446).

Whole-blood iCa measurements (assayed using Horiba LAQUAtwin Ca-11C) and total serum calcium measurements (assayed using Indiko Thermo Fisher Scientific) from 885 blood samples from 102 cows were analyzed, and the association between them is shown in Figure 4. Based on Deming regression, they were moderately associated (R^2^ = 0.4904), and the regression equation was Y = 0.3229X + 0.2841, where the intercept was 0.2841 (95% confidence interval: 0.2351 to 0.3332) and the slope was 0.3229 (95% confidence interval: 0.3011 to 0.3446) Therefore, as the tCa concentration increased, the iCa concentration also increased, although the association was not completely linear.

**Figure 5:**
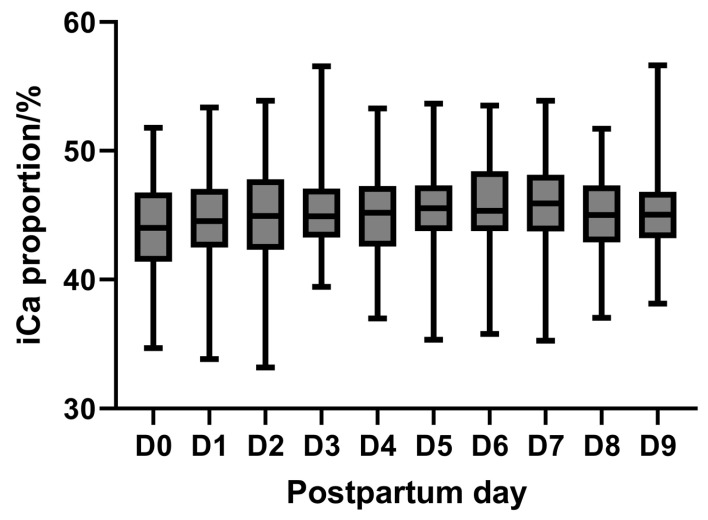
Variations in whole-blood iCa-to-tCa ratio in postpartum dairy cows. Whole-blood iCa concentrations were determined cow-side by Horiba LAQUAtwin Ca-11C, and serum tCa concentrations were determined by Indiko Thermo Fisher Scientific.

In addition, changes in the ratio of blood iCa to tCa in dairy cows during the 9 days after calving were assessed. The ratio of iCa to tCa in dairy cows increased gradually from days 1 to 3 postpartum (43.78–45.42%); then, it remained more stable (44.98–45.86%) from days 3 to 6 and then decreased gradually (45.86–44.99%) (Figure 5). Overall, during the 9 days after calving, the ratio of blood iCa to tCa remained relatively stable at 44.2–47.22%; however, the ratio of blood iCa to tCa varied considerably among cows (ranging from 33.04 to 56.63%). The ranges for whole blood ionized calcium and total serum calcium concentrations are as shown in Table 2.

## 4. Discussion

This study assessed the Horiba LAQUAtwin Ca-11C for measuring whole-blood iCa concentrations in dairy cows in a point-of-care (cow-side) scenario, using the Abbott i-STAT 1 assay as a reference. The i-STAT 1 is a reliable testing tool for measuring iCa in dairy cows [15,16,17]. However, its use on dairy farms is limited by its stringent application temperature requirements (17–30 °C), high cost, and long operating time [17]. The current comparison of the Horiba LAQUAtwin Ca-11C and i-STAT 1 indicated good agreement, with a strong association and consistency (in 246 periparturient Holstein cows), providing evidence that the Horiba LAQUAtwin Ca-11C has potential to replace the i-STAT 1 for measuring iCa in bovine whole-blood samples. In addition, the Horiba LAQUAtwin Ca-11C is easy to operate and well suited for use in point-of-care diagnostics on dairy farms. This instrument should enhance the efficiency and accuracy of monitoring iCa concentrations in dairy cows in clinical practice, providing an effective tool for improving dairy cow health management.

In a second study, blood samples were collected from dairy cows within 9 days after calving, and iCa and tCa concentrations were measured to determine their association and changes in the iCa-to-tCa ratio over time (total of 102 dairy cows and 885 blood samples). The association between iCa and tCa concentrations in dairy cows within 9 days postpartum was only moderate, and the relationship was not linear. We concluded that although iCa concentrations reflected tCa concentrations in dairy cows to some extent, the proportion of iCa to tCa varied significantly among individuals.

The ratio of ionized to tCa in the blood of dairy cows is influenced by several factors [18]. First, calcium metabolism is affected by individual cow differences, including genetics, age, body weight, and health status [19]. Second, the proportion of iCa to tCa changes with physiological state. For example, during the dry period, the cow’s calcium requirement is low, and the proportion of iCa in the blood is relatively stable. In contrast, during early and peak lactation, calcium requirements increase significantly, and the proportion of iCa may increase to meet the high calcium demands of the mammary gland. In addition, a decrease in pH in dairy cows increases the proportion of iCa to tCa in the blood, as an acidic environment attenuates the binding of proteins (e.g., albumin) to calcium ions, increasing iCa concentrations [20]. Finally, hormone concentrations also affect calcium homeostasis, with fluctuations in parathyroid hormone and vitamin D, directly affecting calcium metabolism and the iCa-to-tCa ratio [18]. The present study assessed changes in the ratio of iCa to tCa in the blood of recently calved cows. Future studies should address key factors affecting production, such as cow body condition and health status, to further explore the relationship between the ionic calcium ratio and the physiological status of dairy cows, especially in clinical hypocalcemia, abomasal displacement, and other disorders that alter blood ion concentrations.

Many studies have determined the presence of subclinical hypocalcemia in dairy cows by testing blood tCa concentrations and set thresholds for determining the disease [7]. However, blood iCa concentrations are more informative in terms of assessing cow physiological health and function. In that regard, iCa is critical in maintaining intracellular homeostasis and is involved in key physiological processes such as nerve conduction, muscle contraction, blood coagulation, and the regulation of enzyme activity [18]. In addition, iCa more directly reflects calcium bioavailability, whereas tCa concentrations are affected by serum protein concentrations [21]. For example, when a cow is hypoproteinemic, blood tCa concentration may be lower, but this does not necessarily indicate an abnormal iCa concentration. Therefore, relying on tCa alone may cause misinterpretations. In contrast, the regulation of iCa involves mechanisms such as parathyroid hormone and vitamin D, which can reflect changes in cow metabolism in a more timely manner, especially at critical physiological stages (e.g., prepartum and postpartum [18]). In summary, accurate measurement of iCa concentrations in the blood can provide a more direct, rapid, and accurate reflection of the health status and physiological functions of dairy cows, providing a more reliable basis for appropriate interventions and therapeutic measures.

Excluding physiological circumstances, variations in detection conditions may also affect the detection of calcium ions in the blood. Since CO_2_ escapes quickly after blood is collected, resulting in an increase in pH and thus an increase in bound calcium, we ensured that, when testing iCa in blood using the Horiba LAQUAtwin Ca-11C and Abbott i-STAT 1 devices, the test was completed within 1 h. Furthermore, for testing tCa, whole-blood samples were centrifuged and serum was removed within 1 h and frozen. In addition, it has been reported that a certain concentration of heparin has no significant effect on the measurement of iCa [22]. Therefore, blood sample collection tubes containing equilibrated heparin were used for testing in this study. Furthermore, although all samples were collected and measured within the applicable temperature range of the device, serum samples and whole-blood samples were not kept at the same temperature during the assay, due to unavoidable temperature differences, which may have affected the results. During the detection process, electrolyte imbalances observed in certain postpartum cow diseases, such as clinical hypocalcemia and abomasal displacement, including cation imbalances (e.g., Na^+^, K^+^, and Mg^2+^), may introduce potential biases in iCa measurements by altering the activity of iCa in the sample or affecting electrode response time. Therefore, it is essential to conduct further interference studies to ensure accurate measurement outcomes. In this study, testing conditions were strictly controlled to ensure anaerobic collection of blood samples, standardized preservation, and prompt standardized testing, to minimize the influence of these factors. However, it is still necessary to further validate the stability and accuracy of Horiba LAQUAtwin Ca-11C through long-term and large-scale experiments. In addition, the performance of the Horiba LAQUAtwin Ca-11C was merely verified through a short-term and small-scale experiment in comparison with the Abbott i-STAT 1. Therefore, it would be indispensable to conduct additional long-term and large-scale experiments to further substantiate this.

## 5. Conclusions

This study evaluated the application of a portable ionized calcium (iCa) detection device, Horiba LAQUAtwin Ca-11C, at the point of care (dairy farm setting) through a cross-sectional comparison of three instruments that perform blood calcium detection involving 246 blood samples from 246 dairy cows and 885 blood samples from 102 dairy cows. The device exhibited a high degree of correlation and good consistency with an Abbott i-STAT 1 in blood iCa detection. Hence, we inferred that Horiba LAQUAtwin Ca-11C has the potential to act as a rapid on-farm detection tool for dairy cows. Furthermore, there were significant individual variations in the ratio of iCa to total calcium (tCa) in the blood of postpartum dairy cows. Therefore, future studies should further investigate the individual differences affecting calcium metabolism and their relationship with health status to optimize the health management of dairy cows.

## Figures and Tables

**Table 1:** Key information regarding the three blood calcium testing devices.

Type	Horiba LAQUAtwin Ca-11C	Abbott i-STAT 1	Indiko Thermo Fisher Scientific
Range (mmol/L)	0.1–5.0	0.25–2.50	0.10–5.00
Temperature/°C	5–40	17–30	18–30
Consumables	1.25 and 2.5 mmol/L calibration solutions	CG8+ (stored at 4 °C)	Distilled water
Principle	ISE	ISE	Colorimetry
Calibration	Two-point calibration	/	/

**Table 2:** Whole-blood ionized calcium and total serum blood calcium concentrations at calving (D0) and the next 9 days (D1 to D9) in dairy cows.

		D0	D1	D2	D3	D4	D5	D6	D7	D8	D9
tCa (mmol/L)	Min	0.44	1.03	1.54	1.67	1.68	1.74	1.67	1.51	1.74	1.66
	Max	1.32	2.71	2.66	2.65	2.71	2.68	2.67	2.64	2.66	2.62
	Mean	1.01	2.17	2.17	2.24	2.30	2.27	2.29	2.29	2.28	2.29
iCa (mmol/L)	Min	0.33	0.44	0.74	0.79	0.75	0.79	0.66	0.64	0.76	0.73
	Max	0.57	1.22	1.15	1.18	1.25	1.27	1.32	1.23	1.26	1.17
	Mean	0.45	0.96	0.97	1.01	1.03	1.03	1.04	1.05	1.03	1.03

## Data Availability

Data are contained within the article.
